# Fatty acid desaturation index correlates with body mass and adiposity indices of obesity in Wistar NIN obese mutant rat strains WNIN/Ob and WNIN/GR-Ob

**DOI:** 10.1186/1743-7075-6-27

**Published:** 2009-06-11

**Authors:** Shanmugam M Jeyakumar, Pratti Lopamudra, Suryaprakash Padmini, Nagalla Balakrishna, Nappan V Giridharan, Ayyalasomayajula Vajreswari

**Affiliations:** 1 Biochemistry Division, National Institute of Nutrition, Jamai Osmania, Hyderabad-500 604, India; 2 Statistics Division, National Institute of Nutrition, Jamai Osmania, Hyderabad-500 604, India; 3 National Centre for Laboratory Animal Sciences, National Institute of Nutrition, Jamai Osmania, Hyderabad-500 604, India

## Abstract

**Background:**

Microsomal stearoyl-CoA desaturase1 (SCD1) is the rate limiting enzyme involved in the biosynthesis of monounsaturated fatty acids (MUFAs); palmitoleic (16:1) and oleic (18:1) acid from their respective substrates palmitic (16:0) and stearic (18:0) acids. The ratio of 18:1 to 18:0 has been implicated in the regulation membrane fluidity and function. SCD1 is abundantly expressed in obese humans as well as rodent models. However, no studies have correlated the fatty acid desaturation index (16:1/16:0 and 18:1/18:0), an indicator of SCD1 activity with the markers of obesity in terms of body mass index (BMI) and adiposity index (AI). Therefore, here, we attempted to relate the fatty acid desaturation index with BMI and AI in Wistar NIN-obese mutant rat strains namely, WNIN/Ob and WNIN/GR-Ob (with impaired glucose tolerance).

**Methods:**

For this purpose, 200 days old male 6 lean and 6 obese rats of both strains were taken. Fatty acid composition was analyzed in plasma, various tissues such as liver, white adipose tissues (retroperitoneal, epididymal, omental, and subcutaneous) and brown adipose tissue.

**Results:**

Fatty acid composition data showed significant increase in palmitoleic (16:1) and oleic (18:1) acid levels, which were reflected in increased desaturation index (16:1/16:0 and 18:1/18:0) in plasma and all the tissues of obese rats of both strains, when compared with their respective age and sex-matched lean rats. Further, we found a strong positive correlation between desaturation index, BMI and AI in plasma and most of the tissues analyzed.

**Conclusion:**

So far, plasma Δ^9 ^desaturation index has been well correlated with hypertriglyceridemia and we, by employing two models of obesity namely, WNIN/Ob and WNIN/GR-Ob, have shown Δ^9 ^desaturation index of plasma correlated with physical markers of obesity such as BMI and AI. In conclusion, Δ^9 ^desaturation index may serve as a potential sensitive biochemical marker to assess the degree of obesity and impact of therapeutic/nutritional interventions to combat obesity, along with other indicators.

## Background

Obesity, arising out of modern lifestyle has attained the status of "globesity" and affecting the health of millions of people with various socio-economic consequences. Obesity, a condition caused by imbalanced energy homeostasis, manifests itself as excessive fat deposition and often associated with several morbidities, such as type 2 diabetes, hypertension, coronary heart disease, and certain types of cancers, respiratory complications and osteoarthrites [1]. Microsomal stearoyl-CoA desaturase1 (SCD1) is the rate limiting enzyme involved in the biosynthesis of monounsaturated fatty acids (MUFA); palmitoleoyl and oleoyl-CoAs from their respective substrates palmitoyl and stearoyl-CoAs by Δ^9^-cis desaturation. These MUFAs are major substrates for the synthesis of various lipids such as phospholipids (PL), triglycerides (TG), cholesteryl and wax esters. The ratio of stearic (18:0) to oleic (18:1) acids has been implicated in the regulation of cell growth and differentiation through effects on membrane fluidity and signal transduction [2]. Recent studies have established that SCD1 is the most highly expressed gene in human and rodent models of obesity [3,4]. Further, it has been shown that SCD1 determines the fate of energy, whether it should go for storage or for oxidation [5]. Therefore, it appears that the SCD1 activity may be one of the crucial factors that play a major role in the development of obesity and its associated disorders.

Previously, Attie et al have reported a positive correlation between the SCD1 activity, in terms of desaturation index and plasma TG levels both in humans and mice [6]. Further, another study by Mar-Heyming et al, wherein dyslipidemia has been shown to be positively correlated with plasma desaturase index in patients of familial combined hyperlipidemia [7]. However, so far, no study has related the fatty acid desaturation index to physical markers of obesity such as body mass and adiposity indices (BMI & AI respectively). Therefore, here, we tried to address the relationship between the fatty acid desaturation index, an indicator of SCD1 activity, to BMI and AI of genetically obese rats of Wistar NIN strains namely, WNIN/Ob and WNIN/GR-Ob (with impaired glucose tolerance). The origin, physical and certain biochemical characteristics of these rats strains are already described in our previous publications [8,9].

## Methods

### Animals

200 days old 6 male rats of lean and obese phenotypes of both WNIN/Ob and WNIN/GR-Ob (with impaired glucose tolerance) strains were taken from stock colony fed on standard pellet diet containing 4% ground nut oil (composition of diet is given in table 1). Body weights were recorded. After over-night fasting, blood was collected from retro-orbital sinus for plasma separation. Rats were killed after CO_2 _asphyxiation. Various tissues such as liver, brown adipose tissue (BAT) and several white adipose tissues (WAT) such as retroperitoneal (RPWAT), epididymal (EWAT), subcutaneous (SWAT) and omental (OWAT) were collected, weighed, frozen immediately and stored at -80°C till further analysis.

**Table 1:** Composition of standard pellet diet

**Ingredients**	**g/100**
Wheat flour	22.5
Roasted bengal gram flour	60
Skim milk powder	5
Casein	4
Refined groundnut oil	4
Salt mixture	4
Vitamin mixture	0.5

### Body Mass and Adiposity Indices

Body mass index (BMI) was calculated using the formula; weight in Kg/body length in m^2 ^[8]. Adiposity index was determined by the sum of weights of white adipose tissues divided by bodyweight × 100 and expressed as adiposity percent [8].

### Fast protein liquid chromatography (FPLC)-TG profile

Lipoprotein TG profile of pooled plasma sample (200 μl) was obtained by fast protein liquid chromatography (FPLC)-size fractionation as described earlier [9]. Triglyceride concentration was determined by using commercially available kits (BioSystems, Barcelona, Spain).

### Lipid extraction and Gas Chromatography analysis

Total lipids were extracted from plasma and tissue samples, methylated and analyzed for fatty acid composition by gas liquid chromatography as described earlier [10]. Δ^9 ^desaturation index i.e. ratio of monounsaturated to saturated fatty acids [palmitoleic to palmitic acid (16:1/16:0) and oleic to stearic acid (18:1/18:0) ratios], the indicator of SCD1 activity was calculated.

### Statistics

SPSS software version 15.0 was used for statistical analysis. Results were expressed as means ± SE of 6 animals from each group. The mean values of body weight, BMI and AI of lean and obese rats in different strains were compared by student's t-test. The relationship of desaturation index (16:0/16:1 & 18:0/18:1) of plasma and different tissues with BMI and AI in a given strain was analyzed by Spearman's non-parametric correlation analysis and *P *≤ 0.05 was considered significant.

## Results

### Body weight, AI and BMI

Table 2 shows that obese rats of both WNIN/Ob and WNIN/GR-Ob strains had significantly higher body weight, BMI and AI compared to their respective age and sex-matched lean counterparts.

**Table 2:** Body weight, BMI and AI of WNIN-mutant strain rats

	WNIN/Ob		WNIN/GR-Ob	
	
	Lean	Obese	Lean	Obese
Body weight (g)	314 ± 15	736 ± 22*	433 ± 13	593 ± 22*
BMI	5.4 ± 0.2	10.4 ± 0.3*	6.5 ± 0.1	9.3 ± 0.5*
AI (%)	3.1 ± 0.4	21.8 ± 1.6*	2.5 ± 0.3	16.6 ± 1.0*

Values are means ± SE of 6 rats from each phenotypes. Lean and obese rats of each strain was compared and * denotes statistical significance at *P *≤ 0.05 level (Student's t-test).

### Desaturation Index of plasma and various tissues

**Figure 1:**
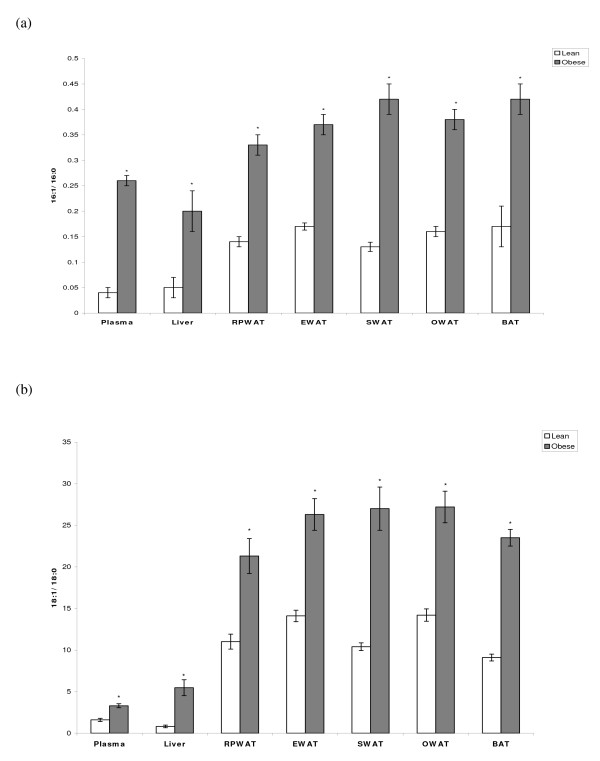
**Desaturation Index of plasma and various tissues of WNIN/Ob strain rats**. Values are mean ± SE of 6 rats from each phenotype. (a) 16:1/16:0 & (b) 18:1/18:0 fatty acid ratio of plasma, liver, various white adipose tissues (WAT) (RPWAT-retroperitoneal, EWAT-epidydimal, SWAT-subcutaneous & OWAT (Omentum) and BAT. *Statistically significant at p ≤ 0.05 level (Student's t-test).

**Figure 2:**
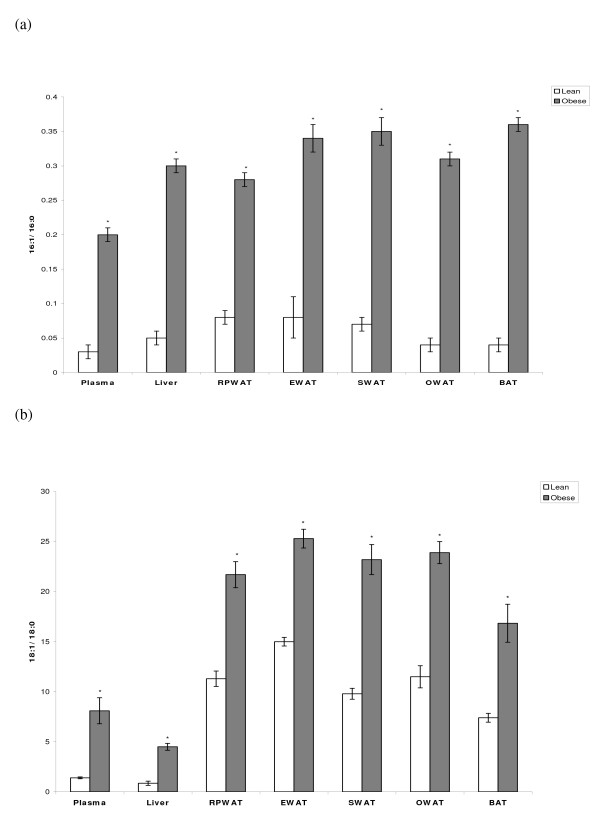
**Desaturation Index of plasma and various tissues of WNIN/GR-Ob strain rats**. Values are mean ± SE of 6 rats from each phenotype. (a) 16:1/16:0 & (b) 18:1/18:0 fatty acid ratio of plasma, liver, various white adipose tissues (WAT) (RPWAT-retroperitoneal, EWAT-epididymal, SWAT-subcutaneous & OWAT-Omentum) and BAT. *Statistically significant at p ≤ 0.05 level (Student's t-test).

Plasma, various white adipose tissues (RPWAT, EWAT, SWAT and OWAT) and BAT of obese rats of both strains showed higher Δ^9 ^desaturation index as evidenced by elevated 16:1 to 16:0 and 18:1 to 18:0 ratios compared to their age and sex-matched lean rats (Figures 1a &1b and 2a &2b).

### Plasma FPLC lipoprotein TG

**Figure 3:**
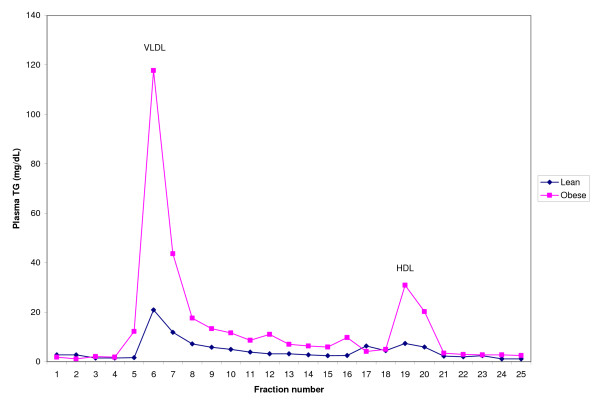
**Plasma-lipoprotein TG profile of WNIN/Ob strain rats by FPLC**. 200 μl of pooled plasma samples from obese and lean rats were size fractionated by FPLC. An aliquot of each fraction was analyzed for TG content. The concentration of TG is expressed as mg/dL plasma.

To authentically confirm the over-expression of hepatic SCD1 led to the hypertriglyceridemia, FPLC-plasma lipoprotein fractionation was done and TG was analyzed. Figure 3. indicates the markedly elevated TG levels in VLDL fraction of obese rats compared to their lean counterparts of WNIN/Ob strain.

### Correlation analysis

Non-parametric spearman's correlation analysis was performed to establish the relationship between the Δ^9 ^desaturation index, BMI and AI. Interestingly, in WNIN/Ob strain, the fatty acid ratio 16:1 to 16:0 of plasma and all the tissues showed a strong positive correlation with BMI and AI.

Further, a significant correlation was also observed for the ratio of 18:1 to 18:0 in plasma, RPWAT, EWAT, OWAT, SWAT and BAT with BMI and AI, while in liver, this ratio did not correlate with these parameters. The correlations are presented in Table 3.

**Table 3:** Correlation between desaturation index, BMI and AI of WNIN/Ob strain rats

WNIN/Ob		16:1/16:0						
		
		Plasma	Liver	RPWAT	EWAT	SWAT	OWAT	BAT
BMI	Correlation coefficient	.698	.680	.675	.888	.888	.875	.729
	
	p value	.025	.031	.032	.001	.001	.001	.017

AI	Correlation coefficient	.638	.754	.636	.879	.879	.794	.636
	
	p value	.047	.012	.048	.001	.001	.006	.048

		18:1/18:0						
		
		Plasma	Liver	RPWAT	EWAT	SWAT	OWAT	BAT

BMI	Correlation coefficient	.809	.590	.837	.778	.778	.760	.839
	
	p value	.005	.073	.005	.008	.008	.011	.002

AI	Correlation coefficient	.842	.539	.867	.903	.903	.758	.842
	
	p value	.002	.108	.002	.000	.000	.011	.002

Spearman's non-paratmetric correlation analysis of 16:1/16:0 and 18:1/18:0 with BMI & AI. *P *≤ 0.05 was considered significant.

Unlike WNIN/Ob, in WNIN/GR-Ob strain, 16:1 to 16:0 ratio in plasma and all the tissue types analyzed significantly correlated with BMI and AI; except OWAT, whose ratio was not correlated with AI. Further, the ratio of 18:1 to 18:0 in plasma, liver, RPWAT, SWAT and OWAT showed strong positive correlation with BMI, while other tissues (EWAT and BAT) failed to show such correlation. On the other hand, this ratio in plasma and various tissues showed a strong positive relationship with AI (Table 4).

**Table 4:** Correlation between desaturation index, BMI and AI of WNIN/GR-Ob strain rats

WNIN/GR-Ob		16:1/16:0						
		
		Plasma	Liver	RPWAT	EWAT	SWAT	OWAT	BAT
BMI	Correlation coefficient	.796	.685	.821	.827	.760	.921	.766
	
	p value	.002	.020	.004	.003	.011	.000	.010

AI	Correlation coefficient	.862	.618	.733	.855	.806	.617	.891
	
	p value	.000	.043	.016	.002	.005	.077	.001

		18:1/18:0						
		
		Plasma	Liver	RPWAT	EWAT	SWAT	OWAT	BAT

BMI	Correlation coefficient	.821	.854	.762	.608	.750	.711	.539
	
	p value	.004	.003	.028	.062	.020	.032	.168

AI	Correlation coefficient	.745	.883	.786	.818	.950	.717	.714
	
	p value	.013	.002	.021	.004	.000	.030	.047

Spearman's non-paratmetric correlation analysis of 16:1/16:0 and 18:1/18:0 with BMI & AI. *P *≤ 0.05 was considered significant.

## Discussion

SCD1 (Δ^9 ^desaturase) is the rate limiting enzyme involved in the biosynthesis of MUFA. In many species, different isoforms of this SCD exist. In mice four isoforms; SCD1, SCD2, SCD3 and SCD4, in rat two isoforms (SCD1, SCD2) and in humans, two SCD isoforms have been identified and characterized [11,5]. SCD1^-/-^-deficient mice have defective hepatic TG and CE synthesis, despite the normal activities of acyl CoA: cholesterol acyltransferase (ACAT) and diacyl glycerol: acyltransferase (DGAT) (the enzymes responsible for cholesterol ester and triglyceride synthesis respectively) and the levels of palmitoleic (16:1) and oleic (18:1) acids in plasma and tissue lipid fractions are lower, whereas palmitic (16:0) and stearic (18:0) acid levels are found to be higher than their wild type controls. These observations suggest that endogenously synthesized MUFAs by SCD1 are the main substrates for the synthesis of hepatic TG and CE [12].

In agreement with the above-mentioned study, earlier, in obese rats of WNIN/Ob strain, we reported significant increase in the fatty acid desaturation index (16:1/16:0 & 18:1/18:0) (both serum and liver), and hypertriglyceridemia, which correlated well with the over-expression of hepatic SCD1mRNA and protein compared to their age and sex-matched lean rats [10]. Similar observations were made even in obese rats of WNIN/GR-Ob strain [9]. Hence, it has been consistently demonstrated that hepatic over-expression of SCD1 results in elevated TG levels and increased desaturation index. In the present study, the fatty acid desaturation index (both 16:1/16:0 & 18:1/18:0) of obese rats was significantly high in plasma, liver, various white adipose tissues and BAT of both WNIN/Ob and WNIN/GR-Ob strains compared to their respective lean mates, which clearly implies that the high expression SCD1 could be responsible for this phenomenon.

Further, to confirm the fact that the SCD1 over-expression leads to hypertriglyceridemia, TG levels in lipoprotein fractions (carried out by FPLC) were estimated. Plasma FPLC profile clearly showed an increase in VLDL-associated TG levels in obese rats compared to their lean counterparts of WNIN/Ob strain. Therefore, the present data unequivocally support that the over-expression of hepatic SCD1 leads to increased TG and VLDL synthesis and secretion and therefore, hypertriglyceridemia. Though, data on plasma FPLC profile on WNIN/GR-Ob strain are lacking, we do expect the similar pattern, as the obese rats of this strain also exhibit hypertriglyceridemia. Interestingly, sterculic acid, a cyclopropane fatty acid has been shown to inhibit 90% of SCD1 enzyme activity due to reduced protein levels, which concomitantly resulted in decreased palmitoleic (16:1) and oleic (18:1) acid levels in cultured differentiating 3T3L1 adipocytes [13]. Therefore, collective evidence from previous studies and the data of the present study on the fatty acid desaturation index (16:1/16:0 & 18:1/18:0) of various tissues of two obese rat strains, clearly suggest the over-expression of SCD1 in obesity.

SCD1 mRNA is highly expressed in white adipose tissue (WAT), brown adipose tissue (BAT), meibomian gland, harderian and preputial glands. This enzyme also serves as a molecular marker for adipocyte differentiation. Some of the recent studies brought out the role of SCD1 as a critical metabolic control point in energy homeostasis, since the activity of this enzyme determines whether the fatty acids should be burnt for energy release or stored as triglycerides [5]. Previously, Cohen et al have demonstrated the crucial role of SCD1 in the regulation energy homeostasis in ob/ob mice. Further, these authors have also shown the repressive effect of leptin on SCD1 expression and therefore, obesity and its associated metabolic changes such as hepatic steatosis in ob/ob mice. Based on these observations, the authors concluded SCD1 as a key player in energy portioning and most of the metabolic effects of leptin are possibly mediated through the suppression of this enzyme [14]. These experimental data unequivocally established SCD1 as one of the potent regulators of energy homeostasis and obesity.

In obese condition, especially in humans, assessing the extent of obesity is very crucial for proper metabolic management during weight loss program. Therefore, relating the anthropometric measures of obesity such as BMI and AI with the biochemical indicators would not only help in assessing the degree of obesity but also the extent of metabolic changes with regard to hypertriglyceridemia and metabolic syndrome. In a population-based cohort study, Waresnjo et al have shown the altered serum fatty acid composition due to SCD1 (Δ^9^), Δ^5 ^and Δ^6^desaturase activities and its association with the metabolic syndrome and thereby suggesting that altered serum fatty acid composition may predict the development of metabolic syndrome [15]. Very recently, in subjects with familial combined hyperlipidemia (FCHL) and fatty acid desaturation index, a significant heritable trait has been correlated with dyslipidemia [7]. Interestingly, in human subjects, positive correlation between the SCD1 activity and hypertriglyceridemia has been reported [6]. In agreement with this, recently, we related the over-expression of hepatic SCD1, hypertriglyceridemia and desaturation index in obese rats of WNIN/Ob strain [10]. In line with previous reports, in the present study, in general, both BMI and AI positively correlated with desaturation index. Further, different tissues showed the correlation at different levels either with BMI or AI and a few of them did not. However, the consistent observation of the present study is that the plasma fatty acid Δ^9 ^desaturation index (both16:1/16:0 and 18:1/18:0) exhibited strong positive correlation with the BMI and AI of rats of both WNIN/Ob and WNIN/GR-Ob strains. Hence, the current data suggest that Δ^9 ^desaturation index of plasma fatty acids is a sensitive biochemical parameter that correlates with BMI and AI, besides hypertriglyceridemia, dyslipidemia and metabolic syndrome.

## Conclusion

In conclusion, plasma fatty acid Δ^9 ^desaturation index; a reflection of SCD1 activity is positively correlated with the BMI and AI in the rats of both WNIN/Ob and WNIN/GR-Ob strains. Therefore, this can be used as a simple sensitive biochemical marker not only to assess the degree of obesity, hypertriglyceridemia, but also to evaluate the efficacy of certain anti-obesity agents.

## Competing interests

The authors declare that they have no competing interests.

## Authors' contributions

SMJ was responsible for animal killing, blood and tissue collection, acquisition of data, analysis and manuscript preparation. PL assisted in plasma FPLC-TG analysis. SP was responsible for fatty acid composition analysis by GC. NB was responsible for the statistical analysis. NVG provided WNIN-mutant strain rats for the study. AV was responsible for the funding, study design, data acquisition, analysis, interpretation & manuscript preparation. All authors read and approved the final manuscript.
